# CB2R Attenuates Intervertebral Disc Degeneration by Delaying Nucleus Pulposus Cell Senescence through AMPK/GSK3β Pathway

**DOI:** 10.14336/AD.2021.1025

**Published:** 2022-04-01

**Authors:** Jiacheng Du, Menglei Xu, Fanchen Kong, Pengfei Zhu, Yubo Mao, Yijie Liu, Hong Zhou, Zhongchen Dong, Zilin Yu, Tong Du, Ye Gu, Xiexing Wu, Dechun Geng, Haiqing Mao

**Affiliations:** ^1^Department of Orthopaedics, The First Affiliated Hospital of Soochow University, Suzhou, China.; ^2^Key Laboratory of Spine and Spinal Cord Injury Repair and Regeneration of the Ministry of Education, Orthopaedic Department of Tongji Hospital, School of Medicine, Tongji University, China.; ^3^Medical college of Soochow University, Suzhou, China.; ^4^Department of Orthopaedics, Changshu Hospital Affiliated to Soochow University, First People’s Hospital of Changshu City, Changshu, China

**Keywords:** CB2R, cellular senescence, IVDD, AMPK/GSK3β

## Abstract

Nucleus pulposus (NP) cell (NPC) senescence is one of the main causes of intervertebral disc degeneration (IVDD). However, the underlying mechanism of NPC senescence is still unclear. The cannabinoid type 2 receptor (CB2R) is a member of the cannabinoid system and plays an important role in antioxidative stress, anti-inflammatory and antisenescence activities. In this study, we used a hydrogen peroxide (H_2_O_2_)-induced NPC senescence model and a rat acupuncture IVDD model to explore the role of CB2R in IVDD in vitro and in vivo. First, we confirmed that the expression of p16INK4a in the NP tissues of IVDD patients and rat acupuncture IVDD models obviously increased accompanied by a decrease in CB2R expression. Subsequently, we found that activation of CB2R significantly reduced the number of SA-β-gal positive cells and suppressed the expression of p16INK4a and senescence-related secretory phenotypes [SASP, including matrix metalloproteinase 9 and 13 (MMP9, MMP13) and high mobility group protein b1 (HMGB1)]. In addition, activation of CB2R promoted the expression of collagen type II (Col-2) and SRY-Box transcription factor 9 (SOX9), inhibit the expression of collagen type X (Col-X), and restore the balance of extracellular matrix (ECM) metabolism. In addition, the AMPK/GSK3β pathway was shown to play an important role in CB2R regulation of NPC senescence. Inhibition of AMPK expression reversed the effect of JWH015 (a CB2R agonist). Finally, we further demonstrated that in the rat IVDD model, the AMPK/GSK3β pathway was involved in the regulation of CB2R on NPC senescence. In conclusion, our experimental results prove that CB2R plays an important role in NPC senescence. Activation of CB2R can delay NPC senescence, restore the balance of ECM metabolism, and attenuate IVDD.

Low back pain (LBP) is the leading cause of global disability and is a heavy burden on the socioeconomic and medical systems [1]. It has been reported that 84% of adults have LBP at least once during their lifetime, while approximately 23% of them will continue to suffer from the work-life challenges caused by LBP [2]. As intervertebral disc degeneration (IVDD) is the main reason for LBP and the incidence rate is increasing year by year, more attention should be given to IVDD [3]. However, the current treatment for LBP is still limited to drugs or surgery to relieve symptoms, and there is no effective treatment for IVDD. The main reason for this is the complex causes of IVDD[4]. Therefore, further studies on the molecular mechanism of IVDD are of great clinical significance.

Aging is one of the main causes that affect IVDD [5]. The quality and levels of extracellular matrix (ECM) anabolism and biomechanical properties decreased, but catabolic processes and senescent cells [6, 7] increase with aging. During IVDD, inflammatory factors released by senescent nucleus pulposus cells (NPCs) stimulate the expression of matrix metalloproteinases (MMPs) and promote the degeneration of the ECM [8]. In addition, the degradation of the ECM leads to a decrease in the water content in the NP, affecting the height of the intervertebral disc and increasing the shear force in the NPCs, eventually leading to the conversion of collagen type II (Col-2) to collagen type I (Col-1), promoting NPC senescence, and aggravating IVDD [9, 10]. Thus, delaying NPC senescence might be an effective method to treat IVDD.

Cellular senescence is an irreversible cell cycle block. It is considered to be one of the important causes of many chronic degenerative diseases, including IVDD[11]. The main signs of cell senescence are the increase in senescence-associated β-galactosidase (SA-β-Gal)-positive cells and the increased expression of cyclin-dependent kinase (CDK) inhibitors (such as p16INK4a)[12]. The inhibition of the CDK-cyclin complex is the main cause of cell proliferation arrest [13]. In addition, senescent cells can also secrete the senescence-associated secretory phenotype (SASP), which affects the microenvironment of the cells and accelerates the senescence of the surrounding cells. SASP mainly includes MMPs and inflammatory factors, such as high mobility group protein b1 (HMGB1) [14]. There are many reasons for cellular senescence, including oxidative stress [15]. Supanji et al. proved that oxidative stress can activate p16INK4a through MAPK, which in turn induces cell senescence [16]. However, the relationship between oxidative stress and cellular senescence remains to be further explored.

Cannabinoid is the main component of the ancient medicinal plant cannabis. Its biological effect is mediated by the cannabinoid type 1 receptor (CB1R) and cannabinoid type 2 receptor (CB2R), two members of the G protein coupled receptor family [17]. Since activation of CB1R has many side effects on the central nervous system, current studies have focused more attention on CB2R [18]. It has been reported that the activation of CB2R inhibits the production of reactive nitrogen and reactive oxygen species (ROS), and then affects neurodegenerative diseases, such as Alzheimer's disease and Parkinson's disease [19, 20]. The role of CB2R in bone and joint diseases has also received much attention [21, 22]. Sophocleous *et al*. demonstrated that CB2R affects bone mass and bone turnover in mice [21]. Similarly, another study showed that CB2R plays a protective role in mouse osteoarthritis [22]. In addition, activation of CB2R can attenuate the senescence of human embryonic kidney cells [23]. However, in the process of IVDD, the relationship between CB2R and NPC senescence requires further investigation.

In this study, we used a hydrogen peroxide (H_2_O_2_)-induced NPC senescence model and a rat acupuncture IVDD model to explore the role of CB2R in IVDD in vitro and in vivo. We found that activation of CB2R can delay NPC senescence, rebalance ECM metabolism, and ultimately attenuate IVDD. Therefore, our research shows that CB2R can attenuate IVDD by delaying NPC senescence via the AMPK/GSK3β pathway.

## MATERIALS AND METHODS

### Ethics statement

The animal experiments required for this study were conducted under the regulation of the Animal Protection and Utilization Committee of the First Affiliated Hospital of Soochow University. This study was approved by the Ethics Committee of the First Affiliated Hospital of Soochow University.

### Experimental animals

Fifty Sprague-Dawley rats (3 months old, 450+50 g) purchased from the animal experiment facility of Suzhou University were used in the animal experiments. The animals were kept at a constant temperature of 21 °C and exposed to light for 12 hours every day.

### Reagents and antibodies

We purchased a CB2R inhibitor (Iodopravadoline, AM630) from Merck (Darmstadt, Germany), a CB2R agonist (JWH015) from APExbio (Houston, USA), an AMPK inhibitor (dorsomorphin 2HCl, Compound C) and an AMPK agonist (acadesine, AICAR) from Selleck (Shanghai, China). In addition, rabbit anti-rat CB2R, cytochrome c oxidase subunit 2 (COX2), nuclear factor, erythroid 2 like 2 (Nrf2), p16INK4α, MMP9, MMP13, HMGB1, Col-2, SRY-Box transcription factor 9 (SOX9), collagen type X (Col-X), adenosine monophosphate-activated protein kinase (AMPK), p-AMPK, glycogen synthase kinase 3 beta (GSK3β), and pS9-GSK3β antibodies, a mouse anti-rat β-actin antibody and goat anti-rabbit IgG antibodies conjugated to Alexa Fluor 647 and Alexa Fluor 488 were obtained from Abcam (Cambridge, UK). Additionally, we purchased a ROS Assay Kit and a SA-β-Gal staining kit from Beyotime (Shanghai, China).

### NPC Culture

Sprague-Dawley rats (6-weeks-old; male) were sacrificed by intraperitoneal injection of an excess of pentobarbital (500 mg/kg). Then, NP tissues from rat caudal intervertebral discs were isolated and digested with 0.5% collagenase type II in a 37 °C water bath for 2 hours. After centrifugation, the precipitate was collected, resuspended and plated in a 6-well plate and incubated with Dulbecco's modified Eagle’s medium/nutrient mixture (DMEM/F-12; Invitrogen) with 15% fetal bovine serum (FBS; Invitrogen, Waltham, MA, USA) and 1% penicillin/streptomycin. The cell plate was placed in an incubator at a constant temperature of 37 °C containing 5% carbon dioxide. When the cell density reached 70-80%, the cells were digested with 0.25% trypsin-ethylenediaminetetraacetic acid (EDTA; Invitrogen). Cells within 3 generations were used in our experiment.

### Cell viability assay

According to the reagent instructions (Dojindo, Rockville, MD, USA), the cell counting kit-8 (CCK-8) was using to access H_2_O_2_ cytotoxicity. First, NPC suspension was added at the concentration of 500 cells/well in 96-well plates. After the cells adhered to the wall, they were mixed with different concentrations of H_2_O_2_ (0, 10, 50, 100, 500, 1000 μM or 0, 50, 75, 100 μM) for 1, 3, 5 days respectively. After incubation, each well was rinsed with phosphate buffered saline (PBS) and added 100 μL DMEM/F-12 (including 10 μL CCK-8 solution). Then, the plate was incubated in a 37 °C incubator for another 2-4 hours. Finally, the absorbance of each well was measured at a wavelength of 450 nm.

### Measurement of ROS level

The appropriate concentration of NPC was inoculated into a 6-well plate with different interventions. After the intervention, an equal volume of 10 μM DCFH-DA was added to each well. The 6-well plate was incubated in a 37 °C incubator for 20 minutes. Then, the cells were digested with trypsin and washed twice with PBS. The cells were then resuspended in PBS containing 2% FBS. Finally, ROS-positive NPCs were detected using flow cytometry.

### SA-β-Gal staining

After the intervention, the plate was incubated with an appropriate amount of SA-β-Gal staining fixative and fixed at room temperature. An appropriate amount of staining solution (Beyotime) was prepared and added to the wells of the plate after washing with PBS, which was then placed in a 37 ? incubator (a CO_2_ incubator was not used) for 24 hours. Finally, images were taken with a high-resolution microscope.

### Reverse transcription quantitative polymerase chain reaction (RT-qPCR)

We extracted total RNA using TRIzol reagent (Invitrogen) and then measured the total RNA concentration using a NanoDrop 2000 (Thermo Fisher Scientific, Waltham, MA, USA). Then, we used a PrimeScript RT Master Mix kit (Takara, Kusatsu, Japan) for reverse transcription, ensuring that the concentration of reverse transcription products reached 1 μg/20 μl, and used the reverse transcription product for amplification. A total of 20 μL of system was used in each reaction, including 10 μL of SYBR Premix Ex TaqTM, 0.5 μL of forward and reverse primers, 1 μL of cDNA and 8 μL of RNase-free H_2_O. The CFX96 Touch real-time PCR detection system (Bio-Rad Laboratories, Hercules, CA, USA) was used for the amplification reaction. The circulation threshold was normalized to the level of glyceraldehyde 3-phosphate dehydrogenase (GAPDH). We quantified the results by using the 2-ΔΔCT method. The specific primers are listed in Table 1.

**Table 1:** Primers based on the rat genome used for RT-PCR.

Gene	Forward Primers	Reverse Primers
GAPDH	GCAAGTTCAACGGCACAG	CGCCAGTAGACTCCACGAC
CB2R	TGAGGAGAGGCGTAGGCA	TCCCGCCATGGACAGACA
p16INK4α	GATAGACTAGCCAGGGCAGC	GAGCTGCCACTTTGACGTTG
Col-2	GAGTGGAAGAGCGGAGACTACTG	CTCCATGTTGCAGAAGACTTTCA
SOX9	TCCCCGCAACAGATCTCCTA	AGCTGTGTGTAGACGGGTTG
Nrf2	TAGATGACCATGAGTCGCTT	CTGTAACTCGGGAATGGAAA
COX2	AAAGGTGTCAGGCAGAAGGG	GGGTGGGCTTCAGCAGTAAT
HMGB1	GTGCCTCGCGGAGGAAAA	TCCCGGCAGGTTTGCAC
MMP9	CTACACGGAGCATGGCAACGG	TGGTGCAGGCAGAGTAGGAGTG
MMP13	TCCATCCCGAGACCTCATGT	AGCATCATCATAACTCCACACG

### Western blot assay

RIPA buffer supplemented with phenylmethanesulfonyl fluoride (PMSF) (Beyotime) was used to obtain the total protein of NPCs cultured in 6-well plates. The protein concentration was quantified by a BCA protein assay kit (Beyotime). Sodium dodecyl sulfate-polyacrylamide gel electrophoresis was used to separate equivalent amounts of protein (20 μg), which were then transferred to a polyvinylidene fluoride membrane (Bio-Rad Laboratories, Hercules, CA USA). Subsequently, the bands were blocked with QuickBlock™ Western Blocking Solution (Beyotime) and incubated overnight with the appropriate primary antibody (anti-β-actin (1:5000, AF5001, Beyotime), anti-CB2R (1:500, ab3561, Abcam), anti-Nrf2 (1:1000, R1312-8, HuaBio), anti-COX2 (1:1000, ab15191, Abcam), anti-p16INK4α (1:1000, ab189034, Abcam), anti-MMP9 (1:1000, ab38898, Abcam), anti-MMP13 (1:5000, ab39012, Abcam), anti-HMGB1 (1:1000, ab18256, Abcam), anti-Col-2 (1:5000, ab34712, Abcam), anti-SOX9 (1:5000, ab185230, Abcam), anti-Col-X (1:300, ab58632, Abcam), anti-AMPK (1:1000, ab32047, Abcam), anti-p-AMPK (1:1000, ab133448, Abcam), anti-GSK3β (1:5000, ab32391, Abcam), or anti-pS9-GSK3β (1:1000, ab131097, Abcam)). Then, the secondary antibody was added and incubated for one hour, followed by detection with an electrochemiluminescence reagent (Thermo Fisher Scientific). Image Lab 3.0 software (Bio-Rad Laboratories) was used to quantify the band images.

### Immunocytochemical (ICC) staining

NPCs were plated in a 24-well plate at 10000/ml and treated with different intervention methods. After the intervention, the cells were washed with PBS, fixed with 4% paraformaldehyde, and then permeabilized with Triton. After blocking nonspecific binding sites with fast blocking solution, the cells were incubated with the primary antibody overnight. The next day, the plates were incubated with the secondary antibody for 1 hour and then stained with DAPI for 10 minutes.

### Lentivirus transfection

The lentiviral vector was obtained from gene Pharmaceutical (Shanghai, China). When 30% of NPCs converged, lentiviruses with a multiplicity of infection of 100 were added to the 6-well culture plate. The culture medium was changed after 48 hours. The transfection effect was evaluated by the expression level of CB2R related genes and proteins.

### RNA interference

Gene pharmaceutical company provided siRNA (RNA oligonucleotide). When NPCs reached 60%-70% confluence, diluted siRNA/plasmid and GP transfection partner were added to 6-well plates and cultured in serum-free medium. After 4 hours, the complete medium was added to each well. The transfection effect was evaluated by the expression level of CB2R related genes and proteins.

### Surgical procedures

After fasting for 12 hours and water deprivation for 4 hours, 2% (w/V) pentobarbital (100 mg/kg) was injected intraperitoneally for anesthesia. Then, five rats were randomly removed from 50 rats, the seventh and eighth caudal intervertebral discs were located, and puncture was carried out with a No. 21 needle (the puncture needle must penetrate both sides of the intervertebral disc and hold for 30 seconds). Then, 5 rats were randomly taken out as a normal control. Another 40 rats were punctured with 21 g needle. Co7-8 was used as the control group (control group). Then, 5 μl of JWH015 or AM+JWH was injected into Co9-10, and a mixture of JWH015 and Compound C or AM+JWH and AICAR was injected into Co10-11. Co8-9 was given the same amount of solvent.

### X-ray and magnetic resonance imaging (MRI) analysis

One week after the puncture, the rats were killed by intraperitoneal injection of chloral hydrate. After euthanasia, we kept the rats in a supine position, and placed their tails on the mammography instrument (General Electric Company, USA) for X-ray examination. The parameters were set as follows: collimator 66 cm away from the film, penetration 35 kV, and exposure 63 MA. A 1.5-T system (GE company, USA, repletion time 3000 ms, echo time 80 ms, field of vision 200 mm^2^, scanning thickness 1.4 mm) was used to collect MRI T2 weighted images, and imaging software (DICOM 3.0, China Neusoft PACS/RIS) was used to process them. Then, we used the software to measure and calculate the IVD height index of rats. Image J software 2.1 (Bethesda, MD, USA) was used to analyze the optical density of the IVD.

### Hematoxylin and eosin staining (H&E) and safranin-O staining

The caudal vertebrae of rats were immersed in 10% formalin for 2 days for fixation and in 10% EDTA for 45 days for decalcification. Then, the fixed Co7-Co10 samples were embedded in paraffin. H&E and safranin fast green staining were performed on sections with a thickness of 6 µm/sheet. The histological grade was determined according to the method of Masuda after microscope observation.

**Figure 1.**
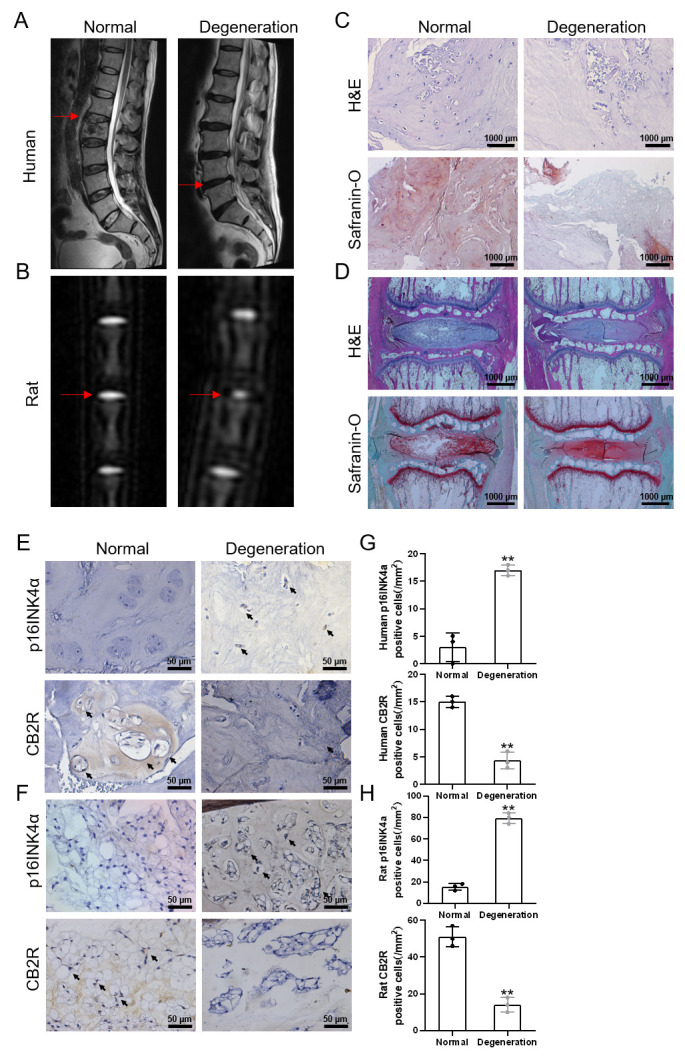
Low expression of CB2R in degenerated IVD tissue. (A) T2-weighted MRI scans of human IVD (red arrows indicate the corresponding IVD); (B) T2-weighted MRI scans of rat caudal IVD at 1 week after operation. (red arrow indicate the corresponding IVD); (C) H&E and safranin-O staining of human NP tissues (scale bar = 1000 μm); (D) H&E and safranin-O staining of rat NP tissues (scale bar = 1000 μm). (E-F) Immunohistochemical staining results of CB2R and p16INK4a in human and rat NP tissues (scale bar = 50 μm, black arrows indicate positive cells); (G-H) Quantification of CB2R and p16INK4a expression in the NP tissues of human and rat. Each experiment was conducted thrice. **p < 0.01 vs. the normal group.

### Immunohistochemistry (IHC) staining

The specific expression levels of CB2R, Nrf2, p16INK4α, Col-2, and SOX9 in rat IVD tissue were observed by immunohistochemistry staining. After the sections were dewaxed with xylene and hydrated with gradient ethanol, the nonspecific binding sites were blocked with horse serum. Then, an appropriate volume of primary antibody was added to the surface of the tissue and placed at 4 °C overnight. On the second day, the samples were incubated with secondary antibody and the tertiary antibody at room temperature for 30 minutes, stained with diaminobenzidine, and counterstained with hematoxylin. After the above steps, the relevant pictures were taken under an upright microscope.

### Statistical analysis

All data were analyzed by SPSS 25.0 statistical software, using the mean±standard deviation, a statistical method using one-way analysis of variance (ANOVA), and pairwise comparison between groups using the LSD-t test. P < 0.05 was considered statistically significant. All data were subjected to Shapiro-Wilk test in GraphPad to verify whether our results conform to the normal distribution before performing ANOVA or LSD-t test

**Figure 2.**
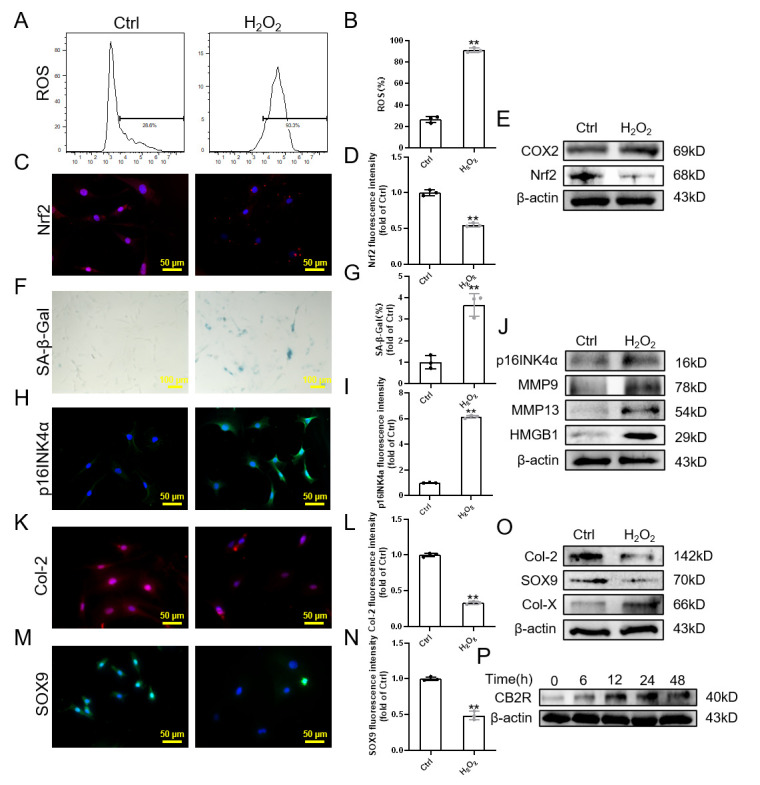
CB2R lowly expressed in H_2_O_2_-induced senescence (A-B) Flow cytometry results of ROS; (C-D) Immunofluorescence staining results of Nrf2 (scale bar = 50 μm); (e) Western blot results of Nrf2, COX2; (F-G) SA-β-Gal staining and quantitative analysis results (scale bar = 100 μm); (H-I) Immunofluorescence staining results of p16INK4a (scale bar = 50 μm); (J) Western blot results of p16INK4a, MMP9,MMP13 and HMGB1; (K-N) Immunofluorescence staining results of Col-2 and SOX9 (scale bar = 50 μm); (O) Western blot results of Col-2, SOX9 and Col-X; (p) Western blot results of dose-dependent expression of CB2R expression under oxidative stress.. Each experiment was conducted thrice, and the data are shown as the mean ± SD. ^**^p < 0.01.

## RESULTS

### The expression of CB2R decreases in degenerated NP tissues

To explore the effect of CB2R in the process of IVDD, normal and degenerated IVD tissues were collected from the clinic. In addition, using a rat acupuncture model as the degeneration group, we obtained normal and degenerated IVD tissues from rats. From the results of the T2-weighted MRI, we clearly found that the signal of the normal IVDs was apparently higher than that of the degenerated IVDs (Fig. 1A-B). H&E and safranin-O staining further confirmed that the histological grade of the degenerated intervertebral disc was higher than that of the normal group (Fig. 1C-D). Subsequently, we performed IHC staining on the specimens and found that the expression of p16INK4a in the degenerated group was higher than that in the normal group. In addition, the increase in p16INK4a was accompanied by a decrease in CB2R expression (Fig. 1E-H). These results suggest that CB2R might influence NPC senescence.

### CB2R is highly expressed in H_2_O_2_-induced NPC senescence

Hereafter, to explore the effect of CB2R on NPC senescence, H_2_O_2_ was used to stimulate NPC senescence and degeneration [24]. CCK-8 results showed that 75 μM H_2_O_2_ has little effect on NPC viability (Supplementary Fig. 1A-B). The intervention time was 24 h. Subsequently, we verified that H_2_O_2_ could induce oxidative stress in NPCs (Fig. 2A-E, Supplementary Fig. 1C-D). In addition, it can be seen from Figures 2F-G that the proportion of SA-β-gal positive cells in the H_2_O_2_ group was 3.66-fold that in the control group, which indicates that there were more senescent cells in the H_2_O_2_ group. Consistent with this result, the expression of p16INK4a and SASP (MMP9, MMP13, HMGB1) in the H_2_O_2_ group increased (Fig. 2H-J, Supplementary Fig. 1E-F). These results confirmed that H_2_O_2_ can induce NPC senescence. In addition, the fluorescence intensity of Col-2 and SOX9 decreased obviously in the H_2_O_2_ group (Fig. 2K-N). The expression of Col-2 and SOX9 was also apparently decreased, while the expression of Col-X was considerably increased under the H_2_O_2_ treatment (Fig. 2O, Supplementary Fig. 1G-H). This indicates that H_2_O_2_ affects the metabolic balance of the ECM in NPCs, specifically manifested as a reduction in anabolism and the most powerful catabolism. In addition, western blot results confirmed that the expression of CB2R increased in a time-dependent manner within 24 hours, peaked at 24 hours, and then decreased (Fig. 2Q, Supplementary Fig. 1I). Meanwhile, RT-PCR results showed that CB2R expression was distinctly higher in NPC treated with H_2_O_2_ (Supplementary Fig. 1J). In conclusion, these results suggest that CB2R plays a protective role in H_2_O_2_ induced senescence of NPCs.

### Regulation of CB2R can influence NPC senescence caused by H_2_O_2_

**Figure 3.**
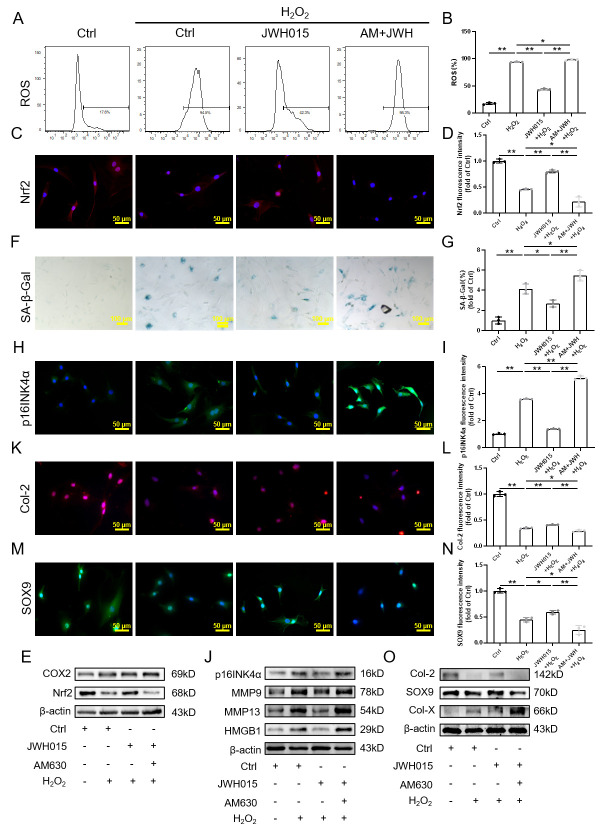
Effect of targeting CB2R on senescence of NPC induced by H_2_O_2_. (A-B) Flow cytometry results of ROS; (C-D) Immunofluorescence staining results of Nrf2 (scale bar = 50 μm); (e) Western blot results of Nrf2, COX2; (F-G) SA-β-Gal staining and quantitative analysis results (scale bar = 100 μm); (H-I) Immunofluorescence staining results of p16INK4a (scale bar = 50 μm); (J) Western blot results of p16INK4a, MMP9,MMP13 and HMGB1; (K-N) Immunofluorescence staining results of Col-2 and SOX9 (scale bar = 50 μm); (O) Western blot results of Col-2, SOX9 and Col-X. Each experiment was conducted thrice, and the data are shown as the mean ± SD. ^*^p < 0.05, ^**^p < 0.01.

To further explore the effect of CB2R on NPC senescence, we used treatment with JWH015 and AM630 to affect CB2R. WB results showed that JWH015 has little effect on the expression of CB2R, and the results of AM630 are the same. The RT-PCR results were consistent with those of the WB (Supplementary Fig. 2A-C). In addition, AM630 has little effect on the oxidative stress level of NPCs treated with H_2_O_2_, while JWH015 reversed the increase in oxidative stress levels induced by H_2_O_2_ (Fig. 3A-E, Supplementary Fig. 2D-G).

As expected, CB2R activation not only inhibited the oxidative stress level but also inhibited NPC senescence. SA-β-gal staining showed that the proportion of senescent cells in the JWH015+H_2_O_2_ group was lower than that in the H_2_O_2_ group, while the addition of AM630 slightly promoted cell senescence, as demonstrated by the higher number of senescent cells than that in the H_2_O_2_ group (Fig. 3F-G). In addition, the expression of p16INK4a and SASP, such as MMP9, MMP13 and HMGB1, was consistent with the SA-β-gal results. Therefore, activation of CB2R can substantially inhibit the senescence of NPCs induced by H_2_O_2_ (Fig. 3H-J, Supplementary Fig. 3A-H).

Moreover, we explored the effect of CB2R on the metabolic balance of the ECM in NPCs. Our results showed that the anabolism of ECM in the JWH015+H_2_O_2_ group was better than that in the H_2_O_2_ group, which was specifically manifested as the enhancement of the immunofluorescence intensity of Col-2 and SOX9. In contrast, the addition of AM630 has little effect on the expression of ECM anabolism-related genes (Fig. 3K-N). The expression of catabolism-related genes in JWH015 +H2O2 decreased. In contrast, the expression of the Col-X gene was higher than that in the H_2_O_2_ group when added AM630 (Fig. 3O, Supplementary Fig. 3I-M).

At the same time, we also transfected NPCs with CB2R overexpressed lentivirus and siRNA, respectively. The results showed that LV-CB2R could significantly promote the expression of CB2R as JWH015, while si-CB2R inhibited the expression of CB2R (Supplementary Fig. 6). si-CB2R1 was chosen for the next experiment. Subsequently, we further confirmed that the use of LV-CB2R could reduce the number of SA-β-gal positive cells, promote the expression of Nrf2 and Col-2 and inhibit the expression of p16INK4a. However, the use of si-CB2R is completely different from the use of LV-CB2R (Supplementary Fig. 7).

**Figure 4.**
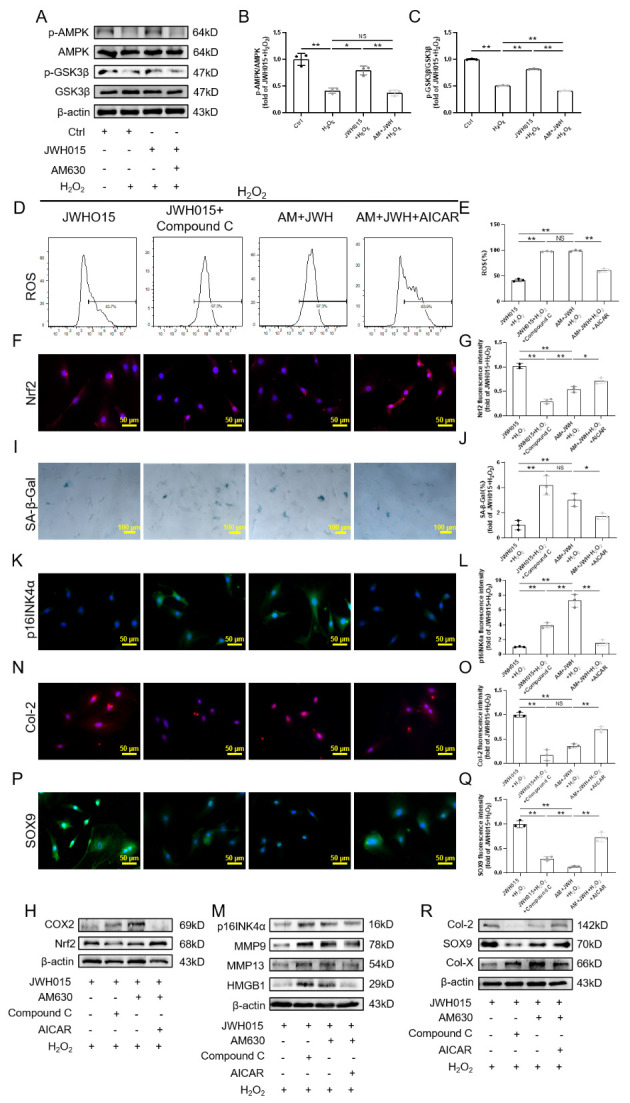
CB2R regulated AMPK/GSK3β pathway plays an important role in H_2_O_2_-induced senescence. (A) Western blot strips of p-AMPK, AMPK, pS9-GSK3β and GSK3β; (B) The ratio of p-AMPK and AMPK protein expression; (C) The ratio of pS9-GSK3β and GSK3β protein expression; (D-E) Flow cytometry results of ROS; (F-G) Immunofluorescence staining results of Nrf2 (scale bar = 50 μm); (H) Western blot results of Nrf2, COX2; (I-J) SA-β-Gal staining and quantitative analysis results (scale bar = 100 μm); (K-L) Immunofluorescence staining results of p16INK4a (scale bar = 50 μm); (M) Western blot results of p16INK4a, MMP9,MMP13 and HMGB1; (N-Q) Immunofluorescence staining results of Col-2 and SOX9 (scale bar = 50 μm); (R) Western blot results of Col-2, SOX9 and Col-X. Each experiment was conducted thrice, and the data are shown as the mean ± SD. ^*^p < 0.05, ^**^p < 0.01.

### The AMPK/GSK3β pathway is involved in the regulation of CB2R in the H_2_O_2_-induced senescence of NPCs

Subsequently, we found that the p-AMPK/AMPK ratio decreased in the H_2_O_2_ group, while the pS9-GSK3β/GSK3β ratio was also decreased. In addition, JWH015 clearly promotes the phosphorylation of AMPK and inhibits the activity of GSK3β. However, the effect of AM630 was the opposite (Fig. 4A-C).

To further explore whether the AMPK/GSK3β pathway plays a role in the effect of CB2R in the H_2_O_2_-induced senescence of NPCs, we used AICAR, a selective agonist of AMPK, and Compound C, a selective inhibitor of AMPK, for the next experiment. Our results showed that Compound C or AICAR did not affect the expression of CB2R even in the presence of JWH015 and AM630 (Supplementary Fig. 4A-C). However, inhibition of AMPK activity considerably antagonized the antioxidant effect of JWH015, while AICAR reversed AM630-induced oxidative stress to some extent (Fig. 4D-H, Supplementary Fig. 4D-G).

**Figure 5.**
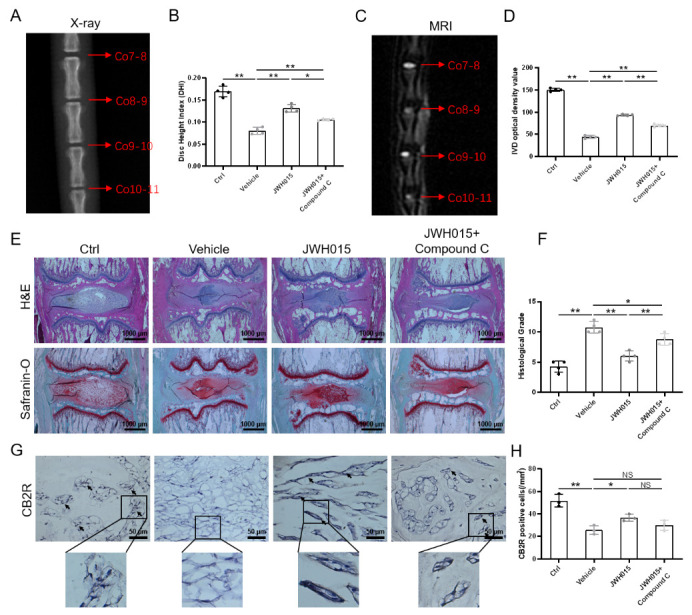
Local intervention of CB2R affects the degeneration of IVDD in rats. (A) X-ray examination of rat caudal at 1 week after surgery. (red arrows indicate different IVDs); (B) Disc height index (DHI) of rat caudal; (C) T2-weighted MRI scans of rat caudal at 1 week after surgery. (red arrows indicate different IVDs); (D) The optical density value of IVD; (E) H&E and safranin-O staining of rat caudal IVD (scale bar = 1000 μm); (F) Histological grade from the H&E and safranin-O staining; (G) Immunohistochemical staining results of CB2R (scale bar = 50 μm, black arrows indicate positive regions or cells); (H) Quantitation of CB2R expression in rat caudal IVD. Each experiment was conducted at least thrice, *p < 0.05, **p < 0.01.

**Figure 6.**
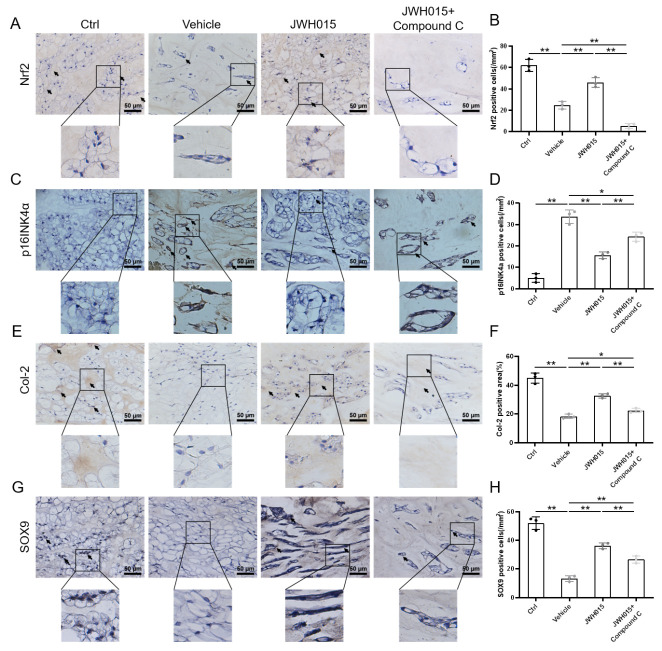
Local intervention of CB2R affects oxidative stress, cellular senescence and ECM anabolism. (A) Immunohistochemical staining results of Nrf2 (scale bar = 50 μm, black arrows indicate positive cells); (B) Quantification of Nrf2 expression in rat caudal IVD; (C) Immunohistochemical staining results of p16INK4a (scale bar = 50 μm, black arrows indicate positive cells); (D) Quantification of p16INK4a expression in rat caudal IVD; (E) Immunohistochemical staining results of Col-2 (scale bar = 50 μm, black arrows indicate positive cells); (F) Quantification of Col-2 expression in rat caudal IVD; (G) Immunohistochemical staining results of SOX9 (scale bar = 50 μm, black arrows indicate positive cells); (H) Quantification of SOX9 expression in rat caudal IVD. ^*^p < 0.05, ^**^p < 0.01.

To further explore whether the AMPK/GSK3β pathway plays a role in the effect of CB2R in the H_2_O_2_-induced senescence of NPCs, we used AICAR, a selective agonist of AMPK, and Compound C, a selective inhibitor of AMPK, for the next experiment. Our results showed that Compound C or AICAR did not affect the expression of CB2R even in the presence of JWH015 and AM630 (Supplementary Fig. 4A-C). However, inhibition of AMPK activity considerably antagonized the antioxidant effect of JWH015, while AICAR reversed AM630-induced oxidative stress to some extent (Fig. 4D-H, Supplementary Fig. 4D-G).

Most importantly, inhibition of AMPK phosphorylation by Compound C reversed the effect of JWH015. In contrast, promoting the phosphorylation of AMPK significantly delayed NPC senescence. In addition, AICAR decreased the expression of p16INK4a and SASP proteins (including MMP9, MMP13 and HMGB1) induced by AM630 (Fig. 4I-M, Supplementary Fig. 5A-H).

In addition, pretreatment with Compound C obviously inhibited the expression of ECM anabolism-related genes (Col-2 and SOX9), while AICAR reversed the decrease in Col-2 and SOX9 expression. WB and RT-PCR results further confirmed this conclusion. In addition, the WB results showed that Compound C significantly promoted the expression of Col-X, while phosphorylated AMPK inhibited the expression of Col-X (Fig. 4N-R, Supplementary Fig. 5I-M). These results prove that CB2R exerts antisenescence effects through the AMPK/GSK3β pathway.

### Activation of CB2R attenuates IVDD via AMPK/GSK3β in vivo

A rat acupuncture model was established to further explore the effect of CB2R on IVDD in vivo. From the X-ray results, we can see that the DHI of the vehicle group decreased by 53.64% (*p* < 0.0001) compared with that of the control group, while that of the JWH015 group increased by 65.15% (*p* = 0.0001) compared with that of the vehicle group. In addition, we found that the JWH015+Compound C group partially reversed the effect of JWH015 (Fig. 5A-B), while AM630 has little effect in IVD height, and local injection of the AICAR reversed the effect of AM630 to a certain extent (Supplementary Fig. 8A-B). In addition, the MRI results were consistent with the X-ray results. From the qualitative MRI results, we found that compared with the control group, the IVD optical intensity of the vehicle group decreased by 70.66% (*p* < 0.0001), while that of the JWH015 group increased by 1.133-fold (*p* < 0.0001) compared with the vehicle group (Fig. 5C-D). AM630 slightly aggravated the loss of water in the NP tissue (Supplementary Fig. 8C-D). These results prove that activation of CB2R can restore disc height and water content in the rat acupuncture model.

In addition, histological staining, such as H&E and safranin-O staining, also demonstrated consistent results with the above images. Through histological staining, we found that in the control group, the NP tissue was relatively complete, showing a large and full oval shape. Acupuncture obviously promotes the degeneration of NP tissue, resulting in the loss of NP tissue, showing a small irregular shape. However, local injection of JWH015 reduced the loss of NP tissue caused by acupuncture, while local injection of AM630 slightly exacerbated the loss of NP tissue (Fig. 5E, Supplementary Fig. 8E). Figure 5F shows that the histological grade of the vehicle group increased by 2.52-fold (*p* < 0.0001) compared with that of the control group, while that of the JWH015 group decreased by 44.12% (*p* = 0.0003) compared with that of the vehicle group. In addition, local injection of JWH015 and Compound C reversed the effect of JWH015. In contrast, local injection of AM630 slightly aggravated the histological grading of the intervertebral disc, while the addition of AICAR reversed this phenomenon (Supplementary Fig. 8F).

Furthermore, IHC staining showed that local injection of JWH015 had little effect on CB2R, while compound C had no effect on CB2R expression. (Fig 5G-H). In contrast, AM630 also has little effect on CB2R, but AICAR has not reversed this phenomenon (Supplementary Fig. 8G-H). This suggests that CB2R may act through the AMPK/GSK3β pathway to attenuate IVDD. Moreover, overexpression of CB2R promoted the expression of Nrf-2, while inactivation of CB2R has little effect on the expression of Nrf-2 (Fig. 6A-B, Supplementary Fig. 9A-B). This suggests that activation of CB2R can reduce the level of oxidative stress during IVDD. We also noted that the number of p16INK4a positive cells in the vehicle group was higher than that in the control group. This phenomenon proves that puncture-induced IVDD accelerates the NPC senescence. Local injection of JWH015 significantly inhibited the expression of p16INK4a, while local injection of AM630 slightly promoted the expression of p16INK4a (Fig. 6C-D, Supplementary Fig. 9C-D). In addition, compared with the control group, the expression of the ECM anabolic index (Col-2 and SOX9) in the vehicle group decreased, while the expression in the JWH015 group was higher than that in the vehicle group, AM630 has little effect on the ECM degradation. In contrast, Compound C reversed the effects of JWH015 against antioxidative stress, antisenescence effect and rebalancing the ECM metabolism (Fig. 6E-H, Supplementary Fig. 9E-H). These results suggest that CB2R is activated through the AMPK/GSK3β pathway to delay IVDD.

## DISCUSSION

**Figure 7.**
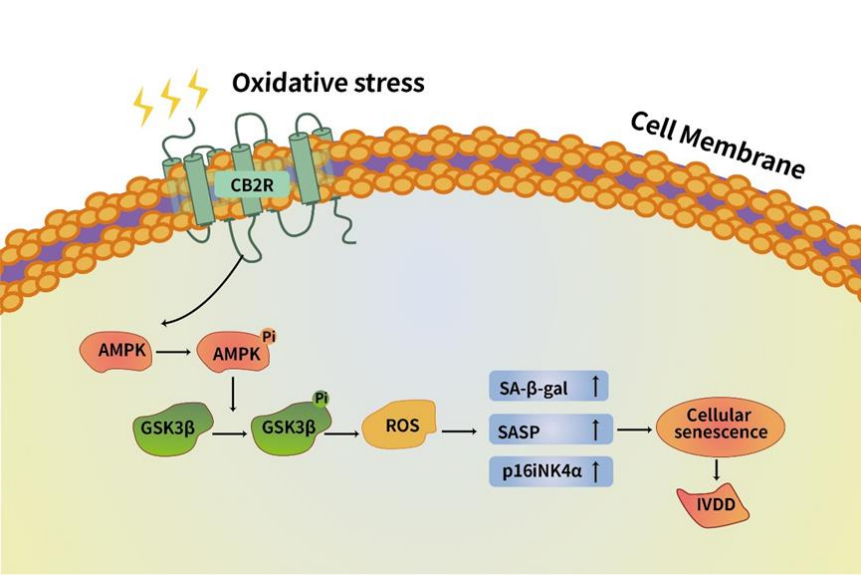
Schematic of protective effects of CB2R. CB2R inhibited the senescence of NPCs and the degeneration of ECM through AMPK/GSKβ pathway under the oxidative damage induced by H_2_O_2_.

Cell senescence plays an important role in IVDD [25]. In this study, we confirmed that activation of CB2R can delay the senescence of NPCs caused by oxidative stress via the AMPK/GSK3β pathway and promote ECM metabolic rebalancing (Fig. 7). In vivo, through an acupuncture rat IVDD model, we confirmed that activation of CB2R can delay the reduction of intervertebral disc height, loss of NP water content, loss of NPC and degradation of ECM caused by acupuncture. This experiment shows that CB2R can be used as an important molecular target to delay or even reverse IVDD.

Cellular senescence refers to the process of change in which the cell proliferation and differentiation abilities and physiological function gradually decline with the passage of time [26]. It is one of the important factors affecting IVDD [27]. In the process of IVDD, the number of senescent cells increases, which promotes the production of SASP, creating an inflammatory microenvironment and promoting the senescence of adjacent cells [28]. In addition, senescent cells also secrete MMPs, such as MMP9 and MMP13, which destroy the metabolic balance of the ECM and accelerate IVDD [29]. In addition, several studies have confirmed that clearing p16INK4a-positive senescent NPCs can significantly improve the metabolic imbalance of NPC and delay the degeneration of IVDD [30, 31].

Oxidative stress is considered to be the main factor leading to cellular senescence and plays an important role in aging-related degenerative diseases (such as cardiovascular disease and cerebrovascular disease) [32]. NP is a kind of tissue with hypoxic conditions and an insufficient blood supply, but its energy supply mainly comes from aerobic metabolism. ROS is its main by-product [33-35]. With the development of IVDD, excessive ROS induces NPC senescence through p53-p21-Rb/p16-RB pathways, inhibits synthesis mechanisms and promotes the production of matrix metalloenzymes [36]. Our experiment showed that H_2_O_2_-induced NPC senescence is accompanied by a large amount of ROS production, which affects the antioxidant levels and influences the expression of Nrf2 and COX2. In addition, H_2_O_2_ can not only increase the level of intracellular ROS, but also cause NPC senescence (as shown by increased in p16INK4a expression, SA-β-gal and SASP), and these two factors leads to an imbalance in ECM metabolism in NPCs.

As an important member of the endocannabinoid system, CB2R plays an important role in a variety of pathological processes (such as inflammation and oxidative stress) [37, 38]. At present, studies have also focused on the effects of the endocannabinoid system on cell senescence [23, 39]. Hu et al. demonstrated that N-stearoyl-l-tyrosine can inhibit H_2_O_2_ induced HEK293/Tau cell senescence by activating CB2R[23]. Through our study, we found that CB2R showed an upward trend within 24 hours and decreased 48h after H_2_O_2_ treatment, which may be due to the increase of CB2R feedback stimulated by H_2_O_2_ to offset the intervention effect of H_2_O_2_ in the short term. Therefore, we further activate or inhibit CB2R to clarify the specific effect of CB2R on IVDD. Our experiment showed that the activation of CB2R could significantly delay the senescence of NPCs induced by H_2_O_2_. In addition, the senescence process of NPC was accompanied by an increase of oxidative stress levels, and the overexpression of CB2R substantially inhibited oxidative stress levels. This suggests that CB2R plays an antisenescence role by restraining the level of oxidative stress. Our results also show that activation of CB2R upregulated the expression of ECM anabolism-related genes (Col-2 and SOX9) and inhibited the expression of ECM catabolism-related genes (Col-X). These results indicate that CB2R plays an antisenescence role and promotes the metabolic balance of the ECM in NPCs.

AMPK is a highly conserved metabolic regulator in evolution [40]. Many studies have proven that AMPK is closely related to cellular senescence and aging-related diseases [41, 42]. However, its effects on cellular senescence mainly rely on its activation of SIRT1, PGC-1α and FOXO [43-45]. The relationship among AMPK, GSK3β and cellular senescence is rarely mentioned. Aimilia D Sklirou et al. proved that 6-bromo-indirubin-3'-oxime (6BIO), a selective inhibitor of GSK3β, has a significant antioxidant effect and can significantly inhibit the senescence of fibroblasts [46]. In addition, lithium, another GSK3β inhibitor, has been shown to delay the aging of flies and *Caenorhabditis elegans* [47]. All of these results suggest that GSK3β is closely related to senescence. Furthermore, Kim et al. proved that activation of AMPK can promote the phosphorylation of GSK3β, inhibit its activity and delay the aging of mice[48]. Most importantly, Wang et al. showed that AMPK activity decreases and GSK3β activity increases in CB2R^-/-^ mice, resulting in tau protein hyperphosphorylation, leading to Alzheimer's disease [49]. This result may explain why CB2R can affect the oxidative stress of NPCs through the AMPK/GSK3β pathway and then affect cellular senescence. Our experimental results showed that CB2R intervention can affect the AMPK/GSK3β pathway. The use of an AMPK inhibitor partially reversed the antioxidant and antisenescence effects and recovery of the ECM metabolic balance of CB2R, while inhibition of AMPK phosphorylation reversed the aggravation of IVDD caused by the decrease in CB2R activity.

## Conclusions

In conclusion, this experiment reveals that the expression of CB2R is decreased in the degenerated IVD tissues. More importantly, activation of CB2R can significantly inhibit H_2_O_2_-induced NPC senescence through AMPK/GSK3β pathway, thereby rebalancing the ECM metabolism of NPCs. In addition, CB2R can also attenuate IVDD in vivo. This study might bring new hope to the treatment of IVDD.

## Supplementary Materials

The Supplementary data can be found online at: www.aginganddisease.org/EN/10.14336/AD.2021.1025.


