# Supplementary material for: CB2R Attenuates Intervertebral Disc Degeneration by Delaying Nucleus Pulposus Cell Senescence through AMPK/GSK3β Pathway
Source: Aging Dis. 2022 Apr 1;13(2):552–67. doi: 10.14336/AD.2021.1025 (PMC8947828; doi:10.14336/AD.2021.1025)
Supplement: Supplementary file 1 [file AD-13-2-552-s.pdf]

SUPPLEMENTARY DATA

CB2R Attenuates Intervertebral Disc Degeneration by Delaying Nucleus Pulposus Cell Senescence through AMPK/GSK3β Pathway

Jiacheng Du[1,*], Menglei Xu [2,*], Fanchen Kong[1,*], Pengfei Zhu[1,*], Yubo Mao[1], Yijie Liu[1], Hong Zhou[1], Zhongchen Dong[1], Zilin Yu[1], Tong Du[3], Ye Gu[4], Xiexing Wu[1], Dechun Geng[1], Haiqing Mao[1]

© 2021. Du J et al. Published online at http://www.aginganddisease.org/EN/10.14336/AD.2021.1025

SUPPLEMENTARY DATA

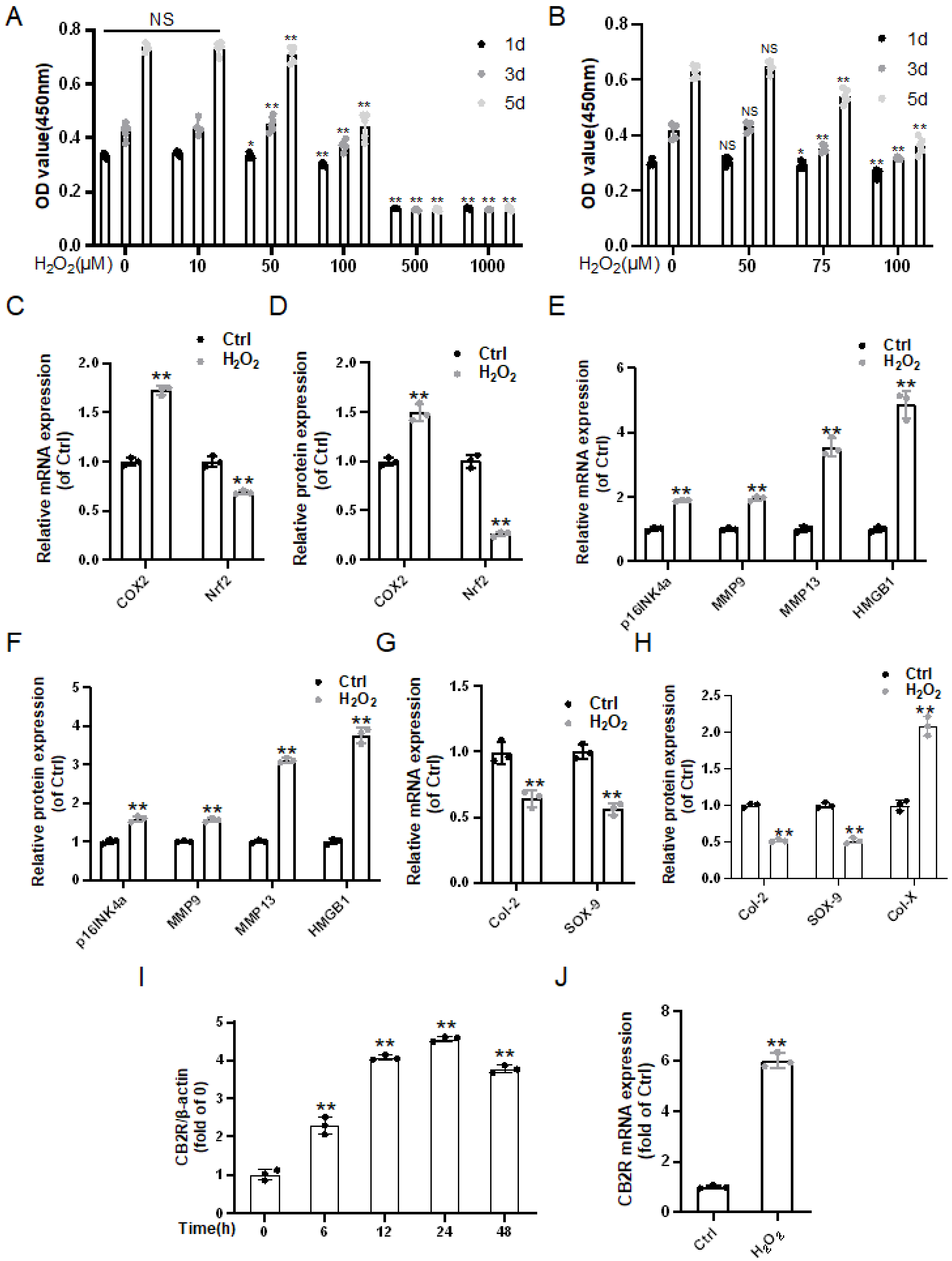

Supplementary Figure 1. (A) Effects of different concentrations (0, 10, 50, 100, 500 and 1000 μM) of H_2O_2 on the proliferation of NPC; **(B)** Effects of different concentrations (0, 50, 75 and 100 μM) of H_2O_2 on the proliferation of NPC; **(C)** RT-PCR results of COX2 and Nrf2; **(D)** WB results of COX2 and Nrf2; **(E)** The mRNA level of p16INK4a, MMP9, MMP13 and HMGB1; **(F)** The protein level of p16INK4a, MMP9, MMP13 and HMGB1; **(G)** RT-PCR results of Col-2 and SOX9; **(H)** Western blot quantitative results of Col-2, SOX9 and Col-X; **(I)** Western blot quantitative results of time-dependent of CB2R expression under oxidative stress; **(J)** RT-PCR results of CB2R. All experiments were performed thrice, and the data are shown as the mean ± SD. $^{*}p < 0.05$, $^{**}p < 0.01$.

© 2021. Du J et al. Published online at http://www.aginganddisease.org/EN/10.14336/AD.2021.1025

SUPPLEMENTARY DATA

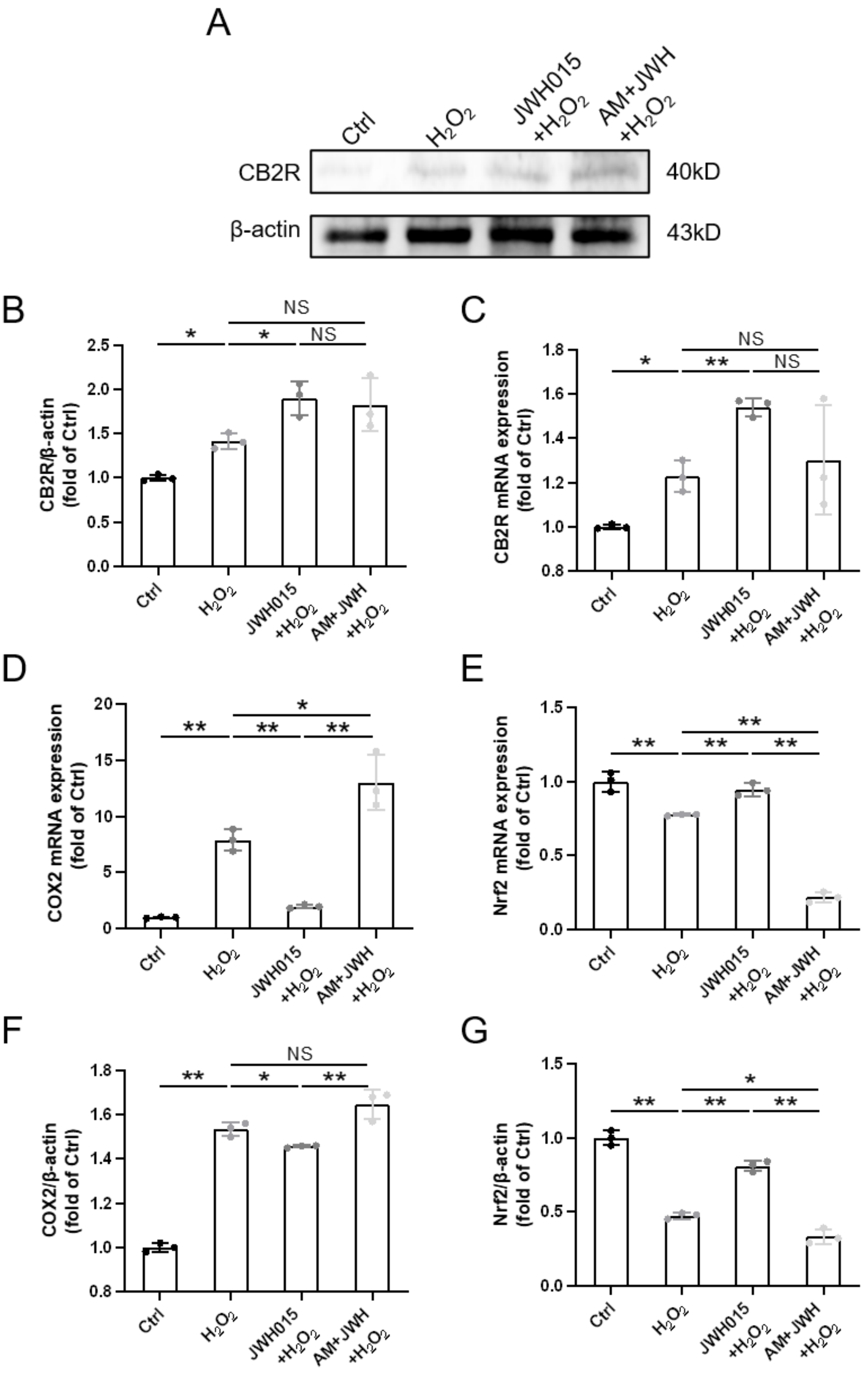

Supplementary Figure 2. (A-B) Western blot results of CB2R; **(C)** RT-PCR results of CB2R; **(D-E)** Western blot quantitative results of COX2 and Nrf2; **(F-G)** The mRNA level of COX2 and Nrf2. All experiments were performed thrice, and the data are shown as the mean ± SD. $^{*}p < 0.05$, $^{**}p < 0.01$.

© 2021. Du J et al. Published online at http://www.aginganddisease.org/EN/10.14336/AD.2021.1025

SUPPLEMENTARY DATA

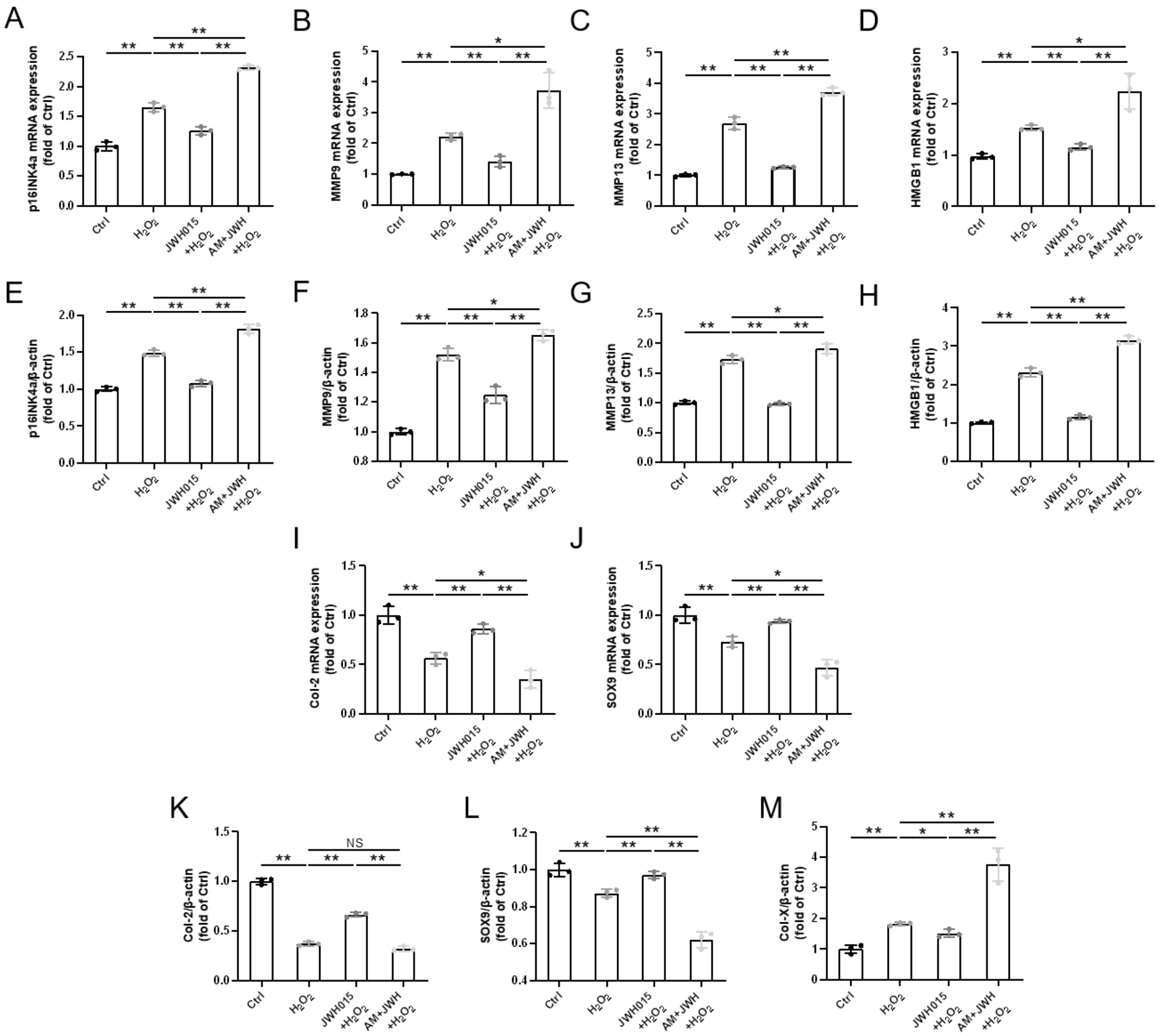

Supplementary Figure 3. (A-H) The mRNA and protein expression levels of p16INK4α, MMP9, MMP13 and HMGB1; **(I-M)** The RT-PCR and western blot results of Col-2, SOX9 and Col-X. All experiments were performed thrice, and the data are shown as the mean ± SD. $^{*}p < 0.05$, $^{**}p < 0.01$.

© 2021. Du J et al. Published online at http://www.aginganddisease.org/EN/10.14336/AD.2021.1025

SUPPLEMENTARY DATA

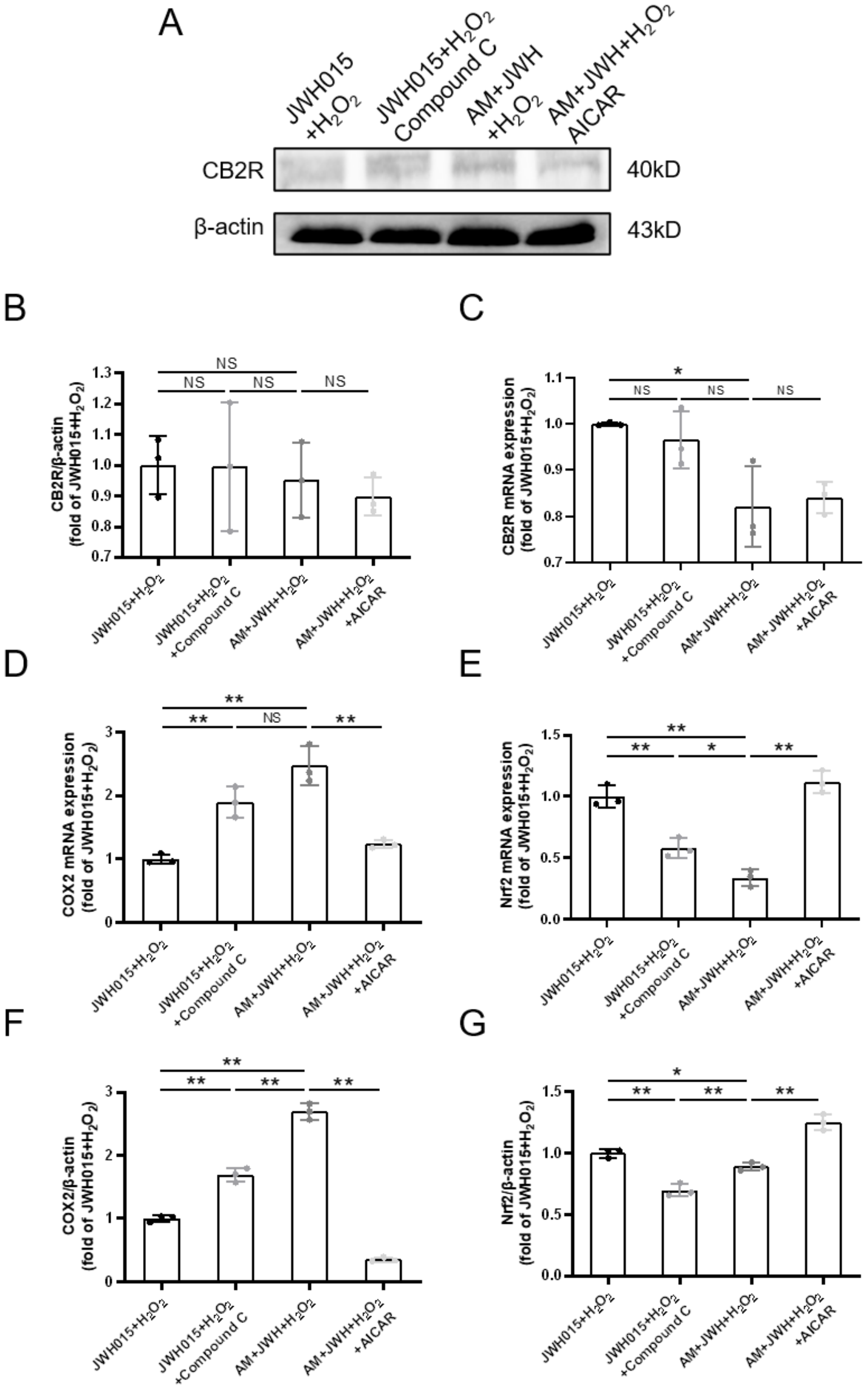

Supplementary Figure 4. (A-B) Western blot results of CB2R; **(C)** RT-PCR results of CB2R; **(D-G)** The mRNA and protein expression levels of COX2 and Nrf2. All experiments were performed thrice, and the data are shown as the mean ± SD. $^{*}p < 0.05$, $^{**}p < 0.01$.

SUPPLEMENTARY DATA

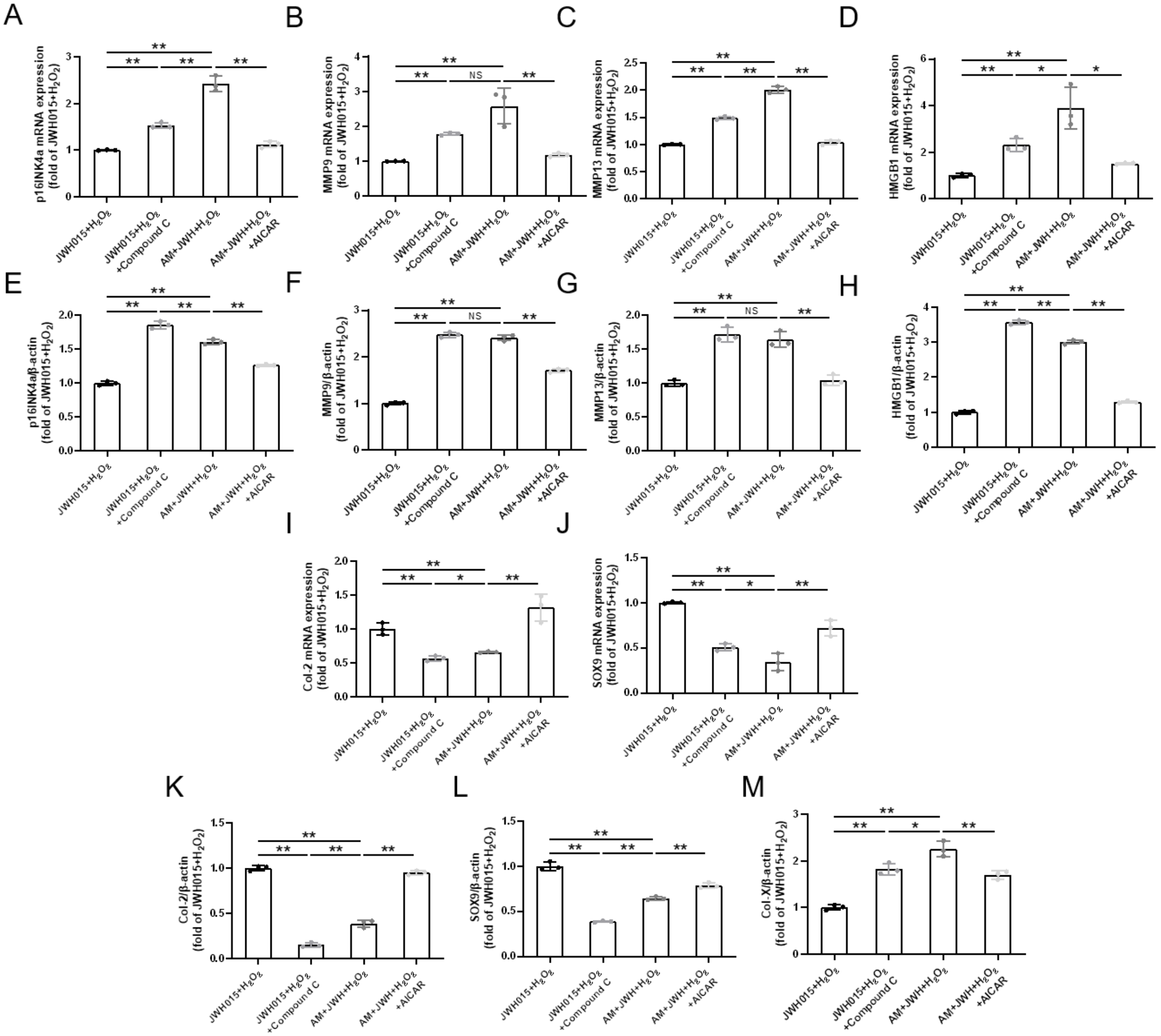

Supplementary Figure 5. (A-H) The mRNA and protein expression levels of p16INK4α, MMP9, MMP13 and HMGB1; **(I-M)** The RT-PCR and western blot results of Col-2, SOX9 and Col-X. All experiments were performed thrice, and the data are shown as the mean ± SD. $^{*}p < 0.05$, $^{**}p < 0.01$.

© 2021. Du J et al. Published online at http://www.aginganddisease.org/EN/10.14336/AD.2021.1025

SUPPLEMENTARY DATA

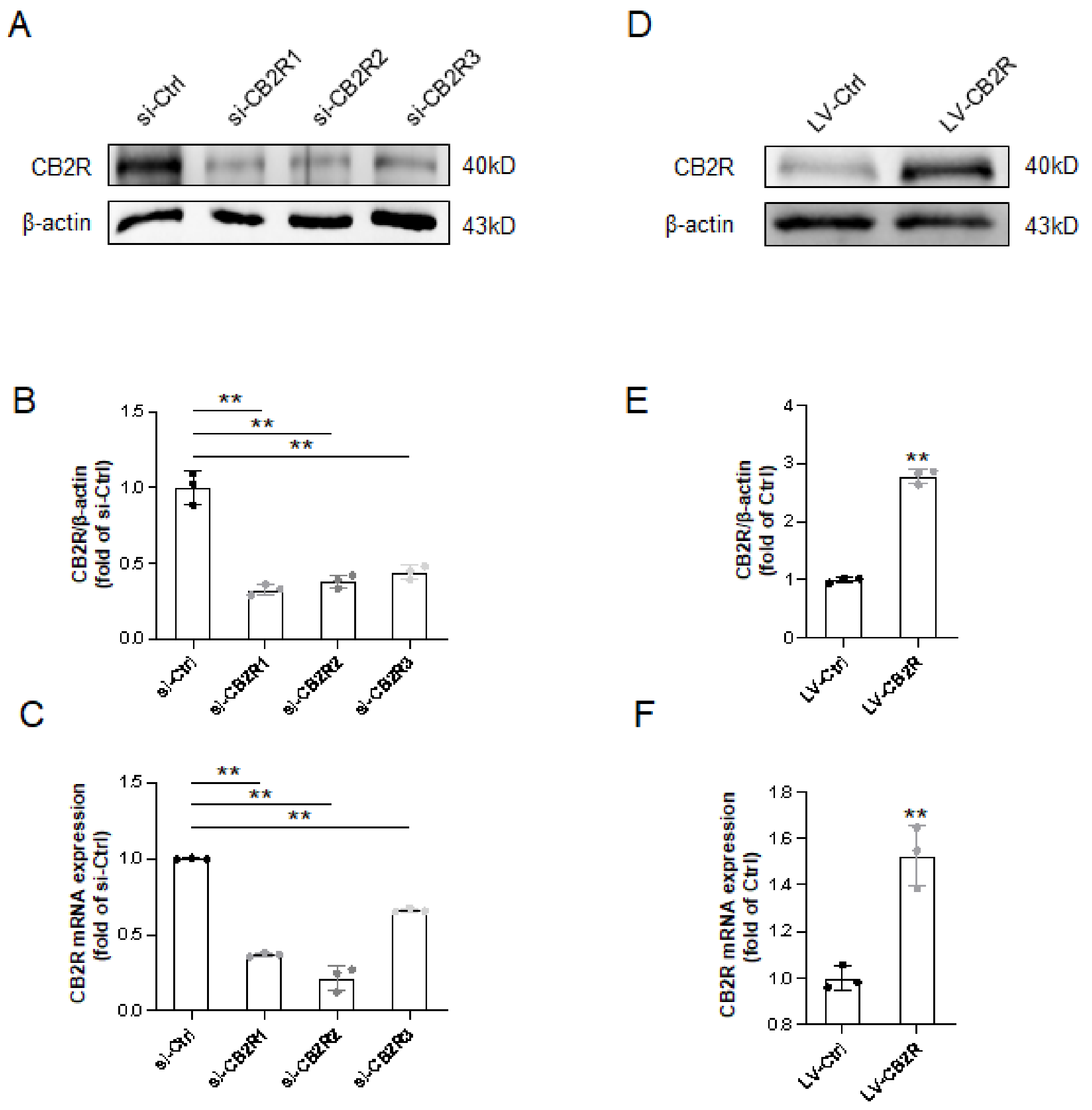

Supplementary Figure 6. (A-C) The western blot and RT-PCR results of CB2R siRNA transfection; **(D-F)** The western blot and RT-PCR results of CB2R lentivirus transfection. All experiments were performed thrice, and the data are shown as the mean ± SD. $^{**}p < 0.01$.

© 2021. Du J et al. Published online at http://www.aginganddisease.org/EN/10.14336/AD.2021.1025

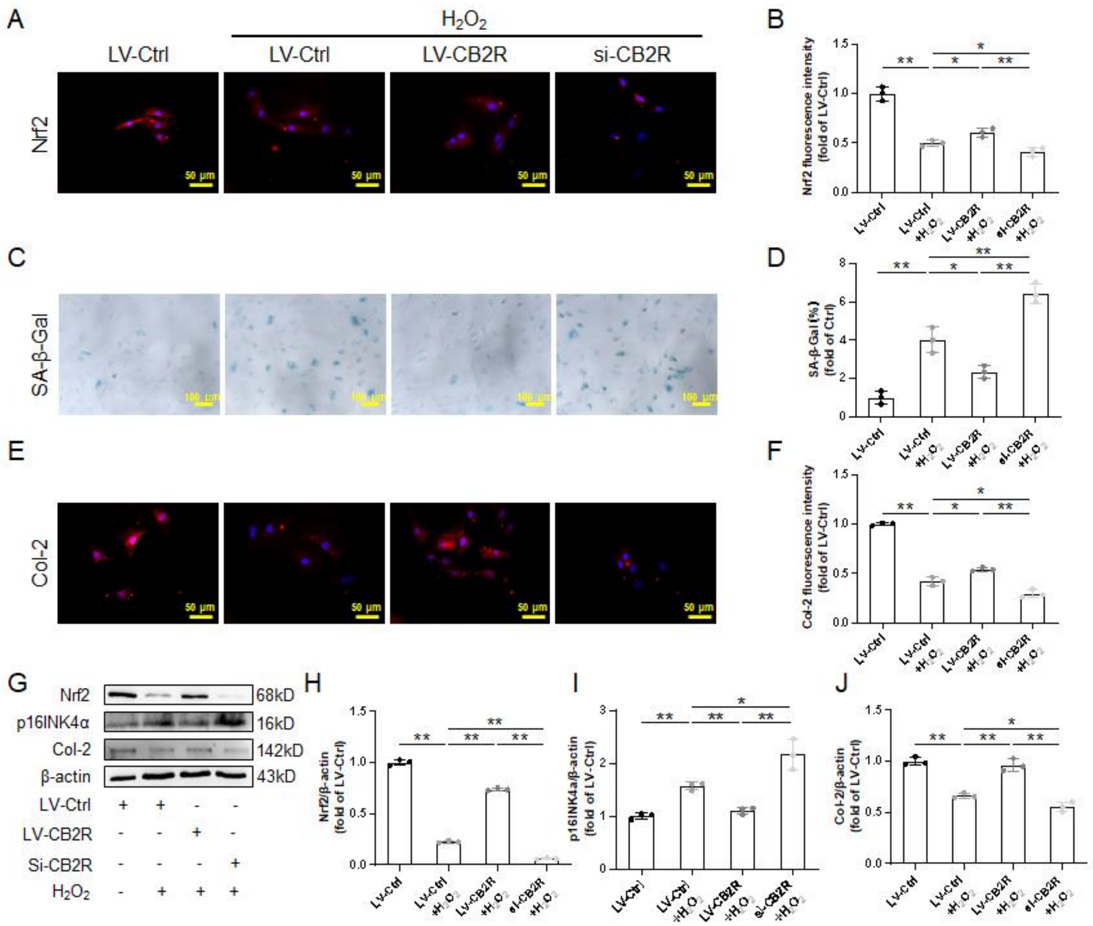

Supplementary Figure 7. (A-B) Immunofluorescence staining results of Nrf2 (scale bar = 50 μm); **(C-D)** SA-β-Gal staining and quantitative analysis results (scale bar = 100 μm); **(E-F)** Immunofluorescence staining results of Col-2 (scale bar = 50 μm); **(G-J)** Western blot results of Nrf2, p16INK4a, Col-2. Each experiment was conducted thrice, and the data are shown as the mean ± SD. **p* < 0.05, ***p* < 0.01.

© 2021. Du J et al. Published online at http://www.aginganddisease.org/EN/10.14336/AD.2021.1025

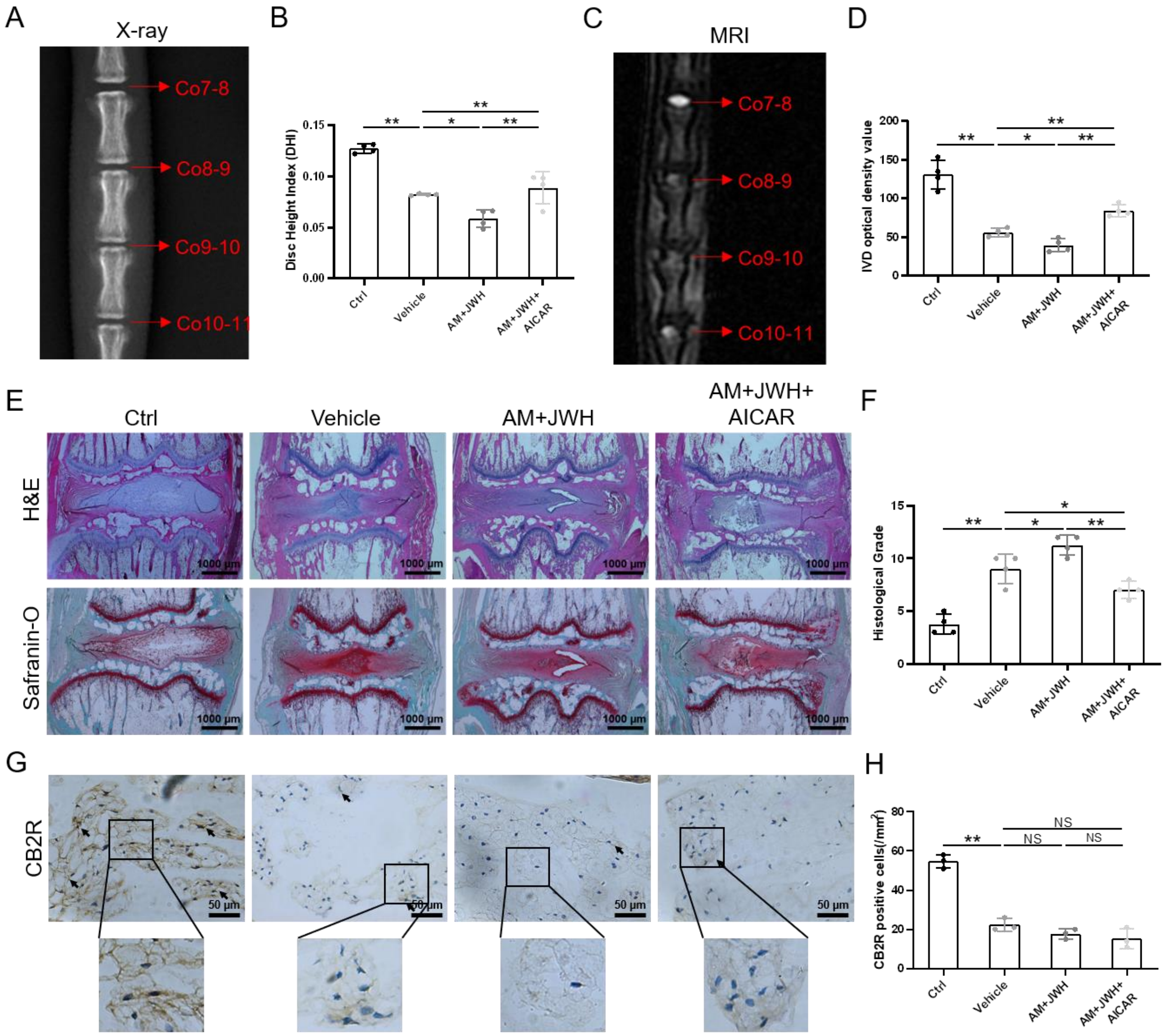

Supplementary Figure 8. (**A**) X-ray examination of rat caudal at 1 week after surgery. (red arrows indicate different IVDs); (**B**) Disc height index (DHI) of rat caudal; (**C**) T2-weighted MRI scans of rat caudal at 1 week after surgery. (red arrows indicate different IVDs); (**D**) The optical density value of IVD; (**E**) H&E and safranin-O staining of rat caudal IVD (scale bar = 1000 μm); (**F**) Histological grade from the H&E and safranin-O staining; (**G**) Immunohistochemical staining results of CB2R (scale bar = 50 μm, black arrows indicate positive regions or cells); (**H**) Quantitation of CB2R expression in rat caudal IVD. Each experiment was conducted at least thrice, *$p < 0.05$, **$p < 0.01$.

© 2021. Du J et al. Published online at http://www.aginganddisease.org/EN/10.14336/AD.2021.1025

SUPPLEMENTARY DATA

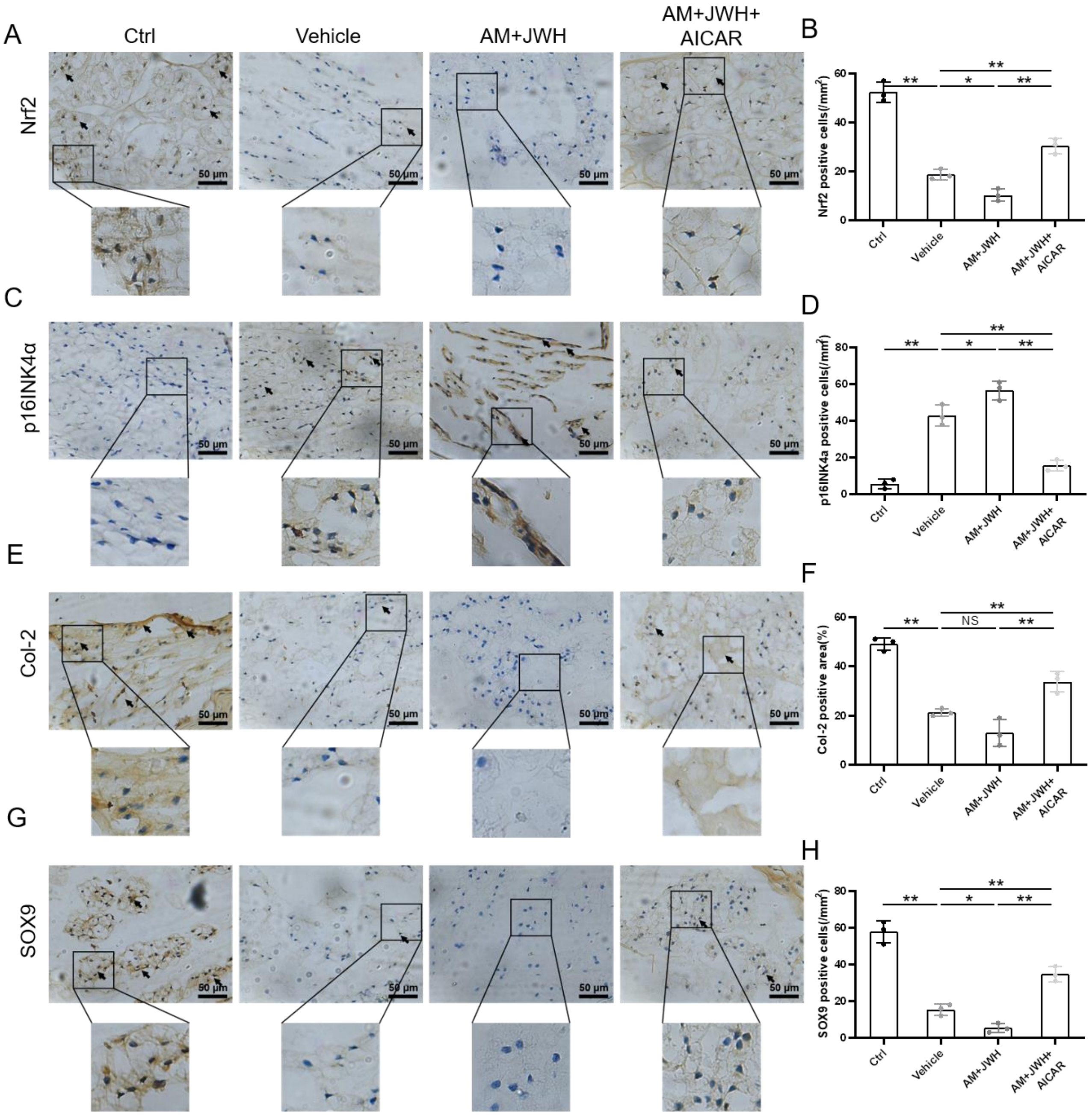

Supplementary Figure 9. (A) Immunohistochemical staining results of Nrf2 (scale bar = 50 µm, black arrows indicate positive cells); **(B)** Quantification of Nrf2 expression in rat caudal IVD; **(C)** Immunohistochemical staining results of p16INK4a (scale bar = 50 µm, black arrows indicate positive cells); **(D)** Quantification of p16INK4a expression in rat caudal IVD; **(E)** Immunohistochemical staining results of Col-2 (scale bar = 50 µm, black arrows indicate positive cells); **(F)** Quantification of Col-2 expression in rat caudal IVD; **(G)** Immunohistochemical staining results of SOX9 (scale bar = 50 µm, black arrows indicate positive cells); **(H)** Quantification of SOX9 expression in rat caudal IVD. $^{*}p < 0.05$, $^{**}p < 0.01$.

© 2021. Du J et al. Published online at http://www.aginganddisease.org/EN/10.14336/AD.2021.1025
